# Comparison of Mechanical Properties of PMMA Disks for Digitally Designed Dentures

**DOI:** 10.3390/polym13111745

**Published:** 2021-05-26

**Authors:** Tamaki Hada, Manabu Kanazawa, Maiko Iwaki, Awutsadaporn Katheng, Shunsuke Minakuchi

**Affiliations:** 1 Gerodontology and Oral Rehabilitation, Graduate School of Medical and Dental Sciences, Tokyo Medical and Dental University, 1-5-45 Yushima, Bunkyo-ku, Tokyo 113-8549, Japan; t.hada.gerd@tmd.ac.jp (T.H.); katheng.gerd@tmd.ac.jp (A.K.); s.minakuchi.gerd@tmd.ac.jp (S.M.); 2 Digital Dentistry, Graduate School of Medical and Dental Sciences, Tokyo Medical and Dental University, 1-5-45 Yushima, Bunkyo-ku, Tokyo 113-8549, Japan; 3 General Dentistry, Graduate School of Medical and Dental Sciences, Tokyo Medical and Dental University, 1-5-45 Yushima, Bunkyo-ku, Tokyo 113-8549, Japan; m.iwaki.gerd@tmd.ac.jp

**Keywords:** CAD/CAM, denture base, flexural strength, discoloration, water sorption, solubility, PMMA

## Abstract

In this study, the physical properties of a custom block manufactured using a self-polymerizing resin (Custom-block), the commercially available CAD/CAM PMMA disk (PMMA-disk), and a heat-polymerizing resin (Conventional PMMA) were evaluated via three different tests. The Custom-block was polymerized by pouring the self-polymerizing resin into a special tray, and Conventional PMMA was polymerized with a heat-curing method, according to the manufacturer’s recommended procedure. The specimens of each group were subjected to three-point bending, water sorption and solubility, and staining tests. The results showed that the materials met the requirements of the ISO standards in all tests, except for the staining tests. The highest flexural strength was exhibited by the PMMA-disk, followed by the Custom-block and the Conventional PMMA, and a significant difference was observed in the flexural strengths of all the materials (*p* < 0.001). The Custom-block showed a significantly higher flexural modulus and water solubility. The water sorption and discoloration of the Custom-block were significantly higher than those of the PMMA-disk, but not significantly different from those of the Conventional PMMA. In conclusion, the mechanical properties of the three materials differed depending on the manufacturing method, which considerably affected their flexural strength, flexural modulus, water sorption and solubility, and discoloration.

## 1. Introduction

Poly(methyl methacrylate) (PMMA) has conventionally been the most common and the is oldest material used for fabricating complete dentures, owing to its advantages, such as excellent dimensional stability in oral environments, low cost, light weight, acceptable aesthetics, and the ease of fabrication and repair [1,2]. It has excellent transparency and aesthetics, which blend into the oral cavity without discomfort. However, there are many concerns related to the use of PMMA, including denture fracture caused by water sorption and impact, as well as the decrease in flexural strength, porosity, and polymerization shrinkage [3,4,5]. Furthermore, the PMMA used in dental materials contains pigments that imitate oral tissue and additives such as nylon or acrylic synthetic fibers in a transparent powder component; the liquid component contains a cross-linking agent and an inhibitor, in addition to the main component, i.e., the methyl methacrylate (MMA) monomer. The nonuniformity of both components when mixing can lead to a decrease in material strength and bacterial invasion, which can decrease biocompatibility [6]. To overcome the shortcomings of PMMA, such as the inadequate mechanical properties and bacterial invasion, the material composition has been changed, and various reinforcing materials have been added [7,8]. However, because the conventional complete denture manufacturing method is complicated, the degree of perfection of the denture may differ depending on the experience and skill of the dentist or dental technician.

In recent years, computer-aided design and computer-aided manufacturing (CAD/CAM) technology has been applied to complete denture treatments. This has helped advance the digitization of denture production, because CAD/CAM technology enables production at a higher speed and accuracy, as well as at a lower cost compared to the manual production of dentures [9,10,11,12]. In the United States, complete dentures fabricated using CAD/CAM and subtractive manufacturing (SM) have been systematized on a commercial basis [13]. The main method for manufacturing dentures using SM involves milling the denture base from a CAD/CAM PMMA disk (PMMA-disk) and then bonding a ready-made artificial tooth into the socket [12].

PMMA-disks are molded under high temperature and pressure in a moisture-free environment and have a highly crosslinked polymer–monomer structure. Therefore, this material has properties superior to those of the material used in the conventional method [14,15,16]. Recently, various PMMA-disks have become commercially available from different manufacturers, but their details have not been disclosed because of copyright issues.

Several studies have investigated the physical properties of PMMA-disks. Al-Dwairi et al. [17] investigated the mechanical properties, including the flexural strength, flexural modulus, and impact strength of PMMA-disks, and they were significantly higher than those of a conventional heat-polymerizing resin (Conventional PMMA); these results indicate the durability of PMMA-disks. The high flexural strength of PMMA-disks is attributed to the voids and porosity generated during the conventional manufacturing process. Steinmassl et al. [18] measured residual monomer release, using liquid chromatography for dentures fabricated using four different CAD/CAM systems, after 7 days of immersion in water. Although the quantity of the residual monomer in the PMMA-disk was exceedingly small, no significant difference was reported compared with that observed in the conventional method.

There are problems in terms of the adhesive strength between the ready-made artificial tooth and the PMMA-disk [19], and the positional accuracy of the artificial tooth [12], when employing the conventional denture manufacturing method that uses SM. Therefore, a PMMA-disk with a two-layer structure was developed; here, the PMMA of the denture base and the artificial tooth were integrated [13]. However, this two-layer PMMA-disk was inferior to ready-made hard resin teeth in terms of its mechanical properties and the aesthetics of the artificial tooth. Furthermore, the current method that involves milling one denture base for each PMMA-disk has disadvantages, such as the high material wastage and long milling time.

**Figure 1:**
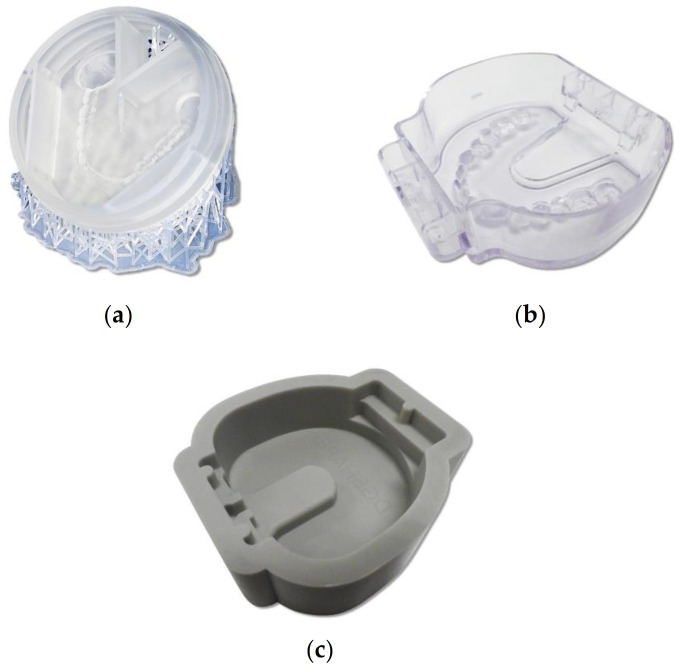
Three types of frames: (**a**) frame manufactured using a 3D printer [20]; (**b**) commercially available frame called the dedicated tray for the denture cutting time reduction kit (CA-DK1-TR; DGSHAPE, Hamamatsu, Japan); and (**c**) commercially available frame called the time reduction kit for cast denture base (CA-DK1; DGSHAPE, Hamamatsu, Japan) [21].

To solve the abovementioned problems, Soeda et al. [20] manufactured a frame into which ready-made artificial teeth could be arranged in advance with a 3D printer; a commercially available self-polymerizing resin can be poured, and the frame can then be used to develop a method for creating custom disks for CAD/CAM. As the inside of this frame can be customized, the amount of self-polymerizing resin that needs to be poured can be minimized. Thus, this frame helps minimize material wastage and significantly decreases the milling time. As shown in Figure 1a, the frame was manufactured using a 3D printer [20]. A frame called the dedicated tray for the denture cutting time reduction kit (CA-DK1-TR; DGSHAPE, Hamamatsu, Japan), which is shown in Figure 1b [21], is commercially available. This frame cuts a tooth mold for fitting artificial teeth into a tray. In addition, as shown in Figure 1c, a commercially available silicone frame time reduction kit for cast denture bases (CA-DK1; DGSHAPE, Hamamatsu, Japan) is made to self-polymerize resin into a block. Using such a frame, dentists and dental technicians can easily polymerize self-polymerizing resin into blocks. Furthermore, the wastage of materials such as PMMA-disk can be reduced and the milling duration shortened, allowing dentures to be manufactured economically and efficiently. However, no studies have evaluated the mechanical and discoloration properties of the self-polymerizing resin that is polymerized into blocks and milled.

Therefore, the present study aimed to compare and examine the flexural strength, flexural modulus, water sorption, solubility, and discoloration of a custom block made from self-polymerizing resin (Custom-block), a commercially available CAD/CAM PMMA disk (PMMA-disk), and heat-polymerizing resin (Conventional PMMA). The null hypothesis was that differences among the three manufacturing methods do not affect the flexural strength, flexural modulus, water sorption, solubility, and discoloration.

## 2. Materials and Methods

### 2.1. Preparation of Various Blocks

**Figure 2:**
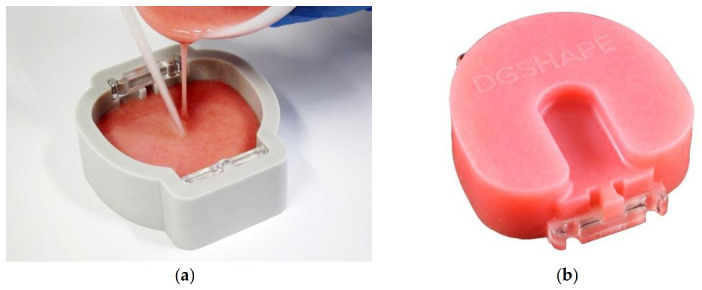
Manufacturing method of the block body. After mixing the self-polymerizing resin (Fit Resin; Shofu) at the standard powder ratio, we poured it into the dedicated tray of the denture cutting time reduction kit (CA-DK1; DGSHAPE): (**a**) pouring technique; (**b**) completed block body.

Table 1 lists the materials used in this study. An acrylic-based self-polymerizing resin (Fitresin; Shofu, Kyoto, Japan) was used to manufacture custom blocks for CAD/CAM. After mixing Fitresin (Shofu) with a standard powder ratio, it was poured into a time reduction kit for cast denture bases (CA-DK1; DGSHAPE, Hamamatsu, Japan) (Figure 2). A suitable denture system (Shofu) that could pressurize and hold resins was used for polymerization; after polymerization at 0.3 MPa and 50 °C for 30 min, according to the manufacturer’s instructions, a block with dimensions of 75 mm × 77 mm × 25 mm was created (Custom-block). Next, a commercially available CAD/CAM PMMA disk (PMMA-disk; Lucitone199 Denture Base Disc; Dentsply Sirona, York, PA, USA) of Φ 98 mm × 30 mm thickness and a heat-polymerizing resin (Acron; GC, Tokyo, Japan) were used. To polymerize the heat-polymerizing resin, it was mixed with a mold made of high-strength type-4 dental stone (NewFujirock; GC, Tokyo, Japan) at a standard powder–liquid ratio; then, the rice-cake-shaped resin was filled into the mold. After pressurization, it was polymerized by heating in a polymerization tank at 78 °C for 8 h, to form a block body (66 mm × 40 mm × 4 mm; Conventional-PMMA). After polymerization, each block was cooled to 23 °C.

### 2.2. Manufacturing the Specimens

We designed each specimen to be slightly larger than the specified size using 3D CAD software (Geomagic Freeform; 3D Systems, Rock Hill, SC, USA) and obtained the output in the STL file format, for cutting the test pieces from each block according to the test item. Each specimen was cut with a milling machine (DWX-52; DG SHAPE, Hamamatsu, Japan). After milling, all specimens were wet-polished using #1200 water-resistant abrasive paper and buffed (Dialap ML150P; Maruto, Tokyo, Japan) with an alumina-based abrasive (particle size: 0.3 µm) for final finishing. The dimensions of the specimen were adjusted according to the ISO standard [22] and confirmed using a digital caliper. Detection force analysis was used to estimate the appropriate sample size based on the results of Prpic et al. [23]; the average flexural strength of self-polymerized PMMA and PMMA-disk were 88.3 ± 10.1 MPa and 104.0 ± 10.4 MPa, respectively. Assuming that the effect size was 1.5 and α = 0.05 and β = 0.95, the required sample size was *n* = 10 (G × Power 3.1.9.4 software: Kiel University, Kiel, Germany).

### 2.3. Three-Point Bending Test

**Figure 3:**
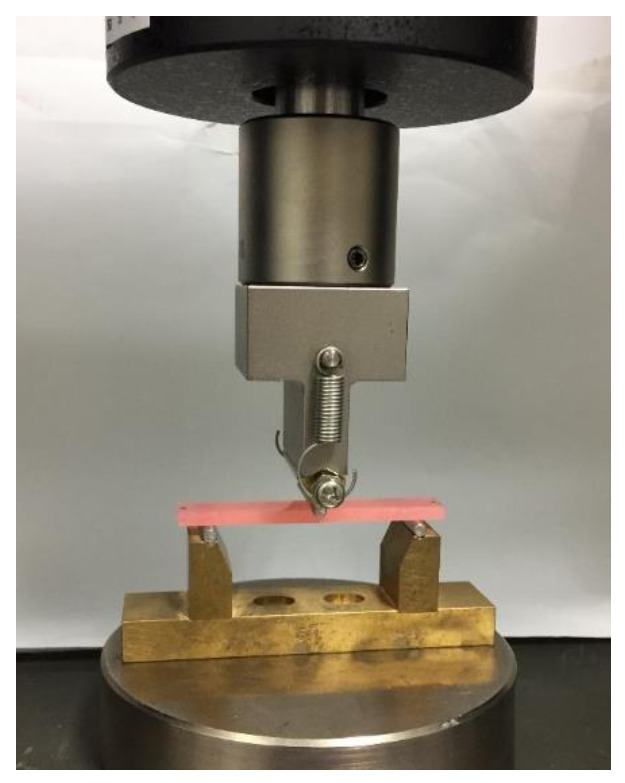
Setup of the three-point bending test. A rectangular specimen measuring 64 mm × 10 mm × 3.3 mm was subjected to a bending test using a universal testing machine until it broke, under a span distance of 50 mm between fulcrums and a crosshead speed of 5 mm/min.

A three-point bending test was performed according to ISO 20795-1: 2013 standards [22]. A rectangular specimen measuring 64 mm × 10 mm × 3.3 mm was used for the bending test (*n* = 10). Each sample was stored in purified water at 37 °C for 50 h. The test was performed with a universal testing machine (AG-Xplus; Shimadzu, Kyoto, Japan) under a span distance of 50 mm and a crosshead speed of 5 mm/min; a load was applied with a load plunger until the sample fractured (Figure 3). The flexural strengths (*FS*, MPa) of the specimens were calculated using

*FS* = 3*Fl*/2*bh*^2^, (1)

where *F*, *l*, *b*, and *h* denote the maximum applied load (N), support span distance (50 mm), specimen width (mm), and specimen height (mm), respectively, prior to testing.

The flexural moduli (*FM*, GPa) of the specimens were calculated using

*FM* = *F*_1_*l*^3^/4*bh*^3^*d*, (2)

where *F*_1_ and *d* denote the load (N) at a point in the straight-line portion of the flexural load–deflection curve and the deflection (mm) at load *F*_1_, respectively.

### 2.4. Water Sorption and Solubility Tests

Water sorption and solubility tests were performed in accordance with ISO 20795-1: 2013 standards [22]. A disk-shaped test specimen of Φ 50 mm × 0.5 mm thickness was used for the test (*n* = 6). To determine the volume (*V*) of each sample, the diameter of the test piece was set as the average of those measured at three points, and the thickness was set as the average of those measured with a digital caliper (MDC-25M; Mitutoyo) at five points (at the center and four points on the outline). The test piece was stored in a desiccator in a constant temperature bath (DX300; Yamato, Tokyo, Japan) at 37 ± 1 °C for 24 h; then, it was weighed using standard-level analytical balances (HR-100AZ; A & D, Tokyo, Japan) to determine the mass after storage in the desiccator at 23 ± 1 °C for 60 min (m1). After the mass became constant within 0.2 mg, the sample was immersed in water at 37 ± 1 °C for 7 days, wiped with a Kimwipe (Kimtech; Kimberly-Clark, Irving, TX, USA), shaken in air for 15 s, and weighed for 60 s after removal from the water. The mass at this time was defined as m2. Subsequently, the mass of the desiccator in a constant temperature bath at 37 ± 1 °C was taken as m3, and the water sorption (*W_sp_*, μg/mm^3^) and solubility (*W_sl_*, μg/mm^3^) were calculated using

*W_sp_* = (m2 − m3) / *V*, (3)

*W_sl_* = (m1 − m3) / *V*. (4)

### 2.5. Staining Test

A staining test was performed with reference to Imamura et al. [24]. A Φ 20 mm × 2.5 mm thick disk-shaped specimen was used for the test (*n* = 7). The immersion liquid was prepared by dissolving 4 g of curry powder (spicy curry powder; S&B Shokuhin Co., Ltd., Tokyo, Japan) in 350 mL of warm distilled water. After storing the test piece in water for one week, the sample was cleaned by washing with water and wiping with Kimwipes; the specimen was stood on a white plate, and the initial color of the test piece was measured using a colorimeter (CR-13; Konica Minolta, Tokyo, Japan; diameter of the measurement area: 8 mm). Next, after completely immersing the specimen in the curry solution at 37 °C for 7 days, the excess curry was flushed with running water, and the water was completely wiped off with a Kimwipe; the color was measured in the same manner as mentioned earlier. The immersion liquid was replaced with fresh liquid every 24 h. The color of each specimen was measured at three locations, and the average was used as the color measurement value (L*, a*, b*; quantified by the colorimetric system) so that the color measurement surface was the same before and after immersion. Based on the values before and after immersion, the discoloration (ΔE*) was calculated as

ΔE* = [(ΔL*)^2^ + (Δa*)^2^ + (Δb*)^2^]^1/2^. (5)

### 2.6. Statistical Analysis

The effects of different polymerization methods on flexural strength, flexural modulus, water sorption, solubility, and discoloration (ΔE*) were compared using statistical analysis software (IBM SPSS statistics 22.0; IBM, New York, NY, USA). Tukey’s multiple comparison test was performed on the obtained average values of flexural strength, flexural modulus, and discoloration (*p* < 0.05). The Kruskal–Wallis test was performed for the average values of water sorption and solubility (*p* < 0.05).

## 3. Results

Table 2 lists the measurement results of the flexural strength, flexural modulus, water sorption, solubility, and discoloration. The flexural strength differed significantly among all materials (*p* < 0.001), and all materials met the requirements of the ISO standard [22]. PMMA-disk showed a significantly higher flexural strength than Custom-block and Conventional PMMA. Furthermore, the flexural modulus significantly differed between Custom-block and PMMA-disk (*p* < 0.001), and between Custom-block and Conventional PMMA (*p* < 0.001); however, no significant difference was observed between the flexural moduli of PMMA-disk and Conventional PMMA (*p* = 0.248). Custom-block showed significantly higher values than the other two materials. Moreover, based on the deflection calculated using Equation (2) and the results presented in Table 2, all the materials broke with a strain of 4% or less, showing brittle fracture characteristics.

The water sorption and solubility met the requirements of the ISO standard [22] for all materials. Water sorption significantly differed between Custom-block and PMMA-disk (*p* = 0.009); however, there was no significant difference between PMMA-disk and Conventional PMMA (*p* = 0.085) or between Custom-block and Conventional PMMA (*p* = 0.150). The water sorption of Custom-block was significantly higher than that of PMMA-disk and Conventional PMMA. Furthermore, the water solubility significantly differed between Custom-block and PMMA-disk (*p* = 0.010), and between Custom-block and Conventional PMMA (*p* = 0.010); however, no significant difference was observed between PMMA-disk and Conventional PMMA (*p* = 0.307). The water solubility of Custom-block was significantly higher than that of PMMA-disk and Conventional PMMA.

**Figure 4:**
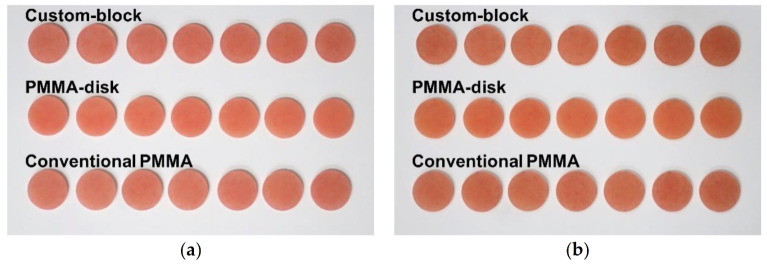
Tendency of discoloration. Discoloration tended to increase from the initial value for 7 days after immersion for all materials. From the top, the samples are of Custom-block, PMMA-disk, and Conventional PMMA. (**a**) Initial value; (**b**) 7 days after immersion.

The discoloration tended to increase from the initial value to 7 days after immersion in all materials (Figure 4). The discoloration after 7 days of immersion significantly differed between Custom-block and PMMA-disk (*p* < 0.001), and between PMMA-disk and Conventional PMMA (*p* < 0.001); however, no significant difference was observed between Custom-block and Conventional PMMA (*p* = 0.901).

## 4. Discussion

We investigated the physical properties of CAD/CAM custom blocks manufactured with the self-polymerizing resin (Custom-block), CAD/CAM PMMA disk (PMMA-disk), and conventional heat-polymerizing resin (Conventional PMMA). The flexural strength, flexural modulus, water sorption, solubility, and discoloration differed significantly under each condition. Thus, our null hypothesis, that the differences between the three different manufacturing methods would not affect the flexural strength, flexural modulus, water sorption, solubility, and discoloration, was rejected.

The mechanical properties of the Custom-block were better than those of the Conventional PMMA. In addition, although the basic physical characteristics of the Custom-block were inferior to those of the PMMA-disk, they met the requirements of the ISO standard [22], which suggested that they could be used in clinical applications. The results of this research are very important for evaluating the effectiveness of the new Custom-block manufacturing system and for expanding the range of material selection.

The flexural properties of the denture base resin are very sensitive to testing conditions, such as the amount of deflection and specimen dimensions. In a previous study, the flexural strength of Lucitone 199 tested in air was 84.84 ± 1.24 MPa [25], whereas that tested in water was 68.5 ± 2.0 MPa [26]. Therefore, when directly comparing the results of this study with those reported previously, the employed testing conditions must be considered. The flexural strength of the PMMA-disk measured in this study was considerably higher than that of Lucitone 199 (84.84 ± 1.24 MPa) determined earlier [25], as well as those of Custom-block and Conventional PMMA. The flexural strength and flexural modulus increased because the generation of internal bubbles was suppressed owing to the ideal heating and pressurization conditions used during the PMMA-disk processing [14,15,16]. Furthermore, the flexural moduli (GPa) of PMMA-disk and Conventional PMMA had no significant differences, but that of Custom-block was significantly higher. The flexural modulus is also called the Young’s modulus (*E*) and can be expressed as

*σ* = *Eε*, (6)

where *σ*, *E*, and *ε* denote the stress, flexural modulus (Young’s modulus), and strain, respectively.

Thus, the higher the flexural modulus, the less easily the material is deformed. Therefore, Custom-block, which is the self-polymerizing resin, may better suppress the deformation and fracture of the denture under occlusal pressure compared to PMMA-disk and the Conventional PMMA. In addition, the values were almost the same as those reported by Iwata [27] (flexural strength: 90.1 ± 4.0 MPa; flexural modulus: 2.9 ± 0.2 GPa), who evaluated the mechanical properties of the same heat-polymerizing resin as the Conventional PMMA in this study. This suggests that the heat-polymerization process in this study was performed appropriately. In this study, the results for Custom-block met the requirements of the Type 1 and Type 2 ISO standards [22] for the flexural strength and flexural modulus, and they showed higher values than those of the control group and those reported in previous studies [28]. Therefore, Custom-block has clinically acceptable mechanical properties and is suitable for fabricating denture bases.

The water sorption of the denture base (polymer material) causes internal stress within the material, because of the stable dimensions; over time, cracks are formed, and the mechanical properties deteriorate [29,30]. Furthermore, the water absorption must be as low as possible, because bacterial flora can cause undesirable odor [31]. Takahashi et al. [32] reported that the mechanical properties of the denture base material decreased as the solubility increased; and therefore, the solubility must also be low.

In this study, the Custom-block exhibited a significantly higher water sorption and solubility than that of PMMA-disk and Conventional PMMA (Table 2). In general, self-polymerizing resins have an excellent dimensional accuracy and operability, because heat shrinkage can be suppressed to lower levels than those possible for heat-polymerizing resins. The high water sorption and solubility of Custom-block is attributed to its low degree of polymerization and high residual monomer [33]. Vallittu et al. [34] revealed that the polymerization temperature and time affect the residual monomer content in the denture base material. In the present study, Custom-block was heat-polymerized at 50 °C for 30 min, and Conventional PMMA was heat-polymerized at 78 °C for 8 h. Furthermore, Custom-block had more monomer units than Conventional PMMA to achieve fluidity [35,36]. Therefore, the water sorption and solubility of Custom-block were higher than those of PMMA-disk and Conventional PMMA.

In addition, the properties of PMMA-disk and Conventional PMMA met the requirements of the ISO standard [22] for Type 1; the water sorption was below 4.3 μg/mm^3^, and the solubility was below 1.1 μg/mm^3^. Furthermore, the properties of Custom-block met the ISO standard [22] for Type 2 (water sorption below 0.9 μg/mm^3^ and solubility below 3.3 μg/mm^3^). Thus, the water sorption and solubility of Custom-block are within the clinically acceptable range for materials used for fabricating denture bases.

Staining indicates that the dye is physically embedded between the molecular lattices of the subject or adsorbed on the surface. A curry solution was used as the staining medium in this study because it produces noticeable staining. Furthermore, measurements conducted using the CIEL* a* b* system are widely used for quantitatively and objectively evaluating the discoloration (ΔE*) of dental materials. However, research on the staining properties of dental materials has mostly focused on crown materials, such as porcelain, hard resin, and cold-curing resin, with few reports on the staining properties of denture base resins [37].

The a* indicates the hue of red in the positive direction, and b* indicates the hue of yellow in the positive direction. In this study, the b* values increased significantly in all specimens 7 days after immersion in the curry solution; this suggests that the hue changed from red to yellow. According to Iwaki et al. [14], the ΔE* value of the heat-polymerizing resin after immersion in the curry solution for one week was 2.46 ± 0.28, and that of the PMMA-disk was 1.61 ± 0.03. The ΔE* values of Conventional PMMA and PMMA-disks used in this study were approximately 2.3 to 2.5 times higher than those reported by Iwaki et al. [14]. In translucent materials such as plastics, incident light penetrates deep inside the specimen, and scattered light is re-emitted as reflected light. Ishikawa [38] reported that if the specimen is thin, it may be affected by the color of the base plate. As the thickness of the specimen in this study was 2.5 mm [39], the color of the base plate could be ignored. Therefore, the ΔE* value obtained in this study was significantly higher than that of the previous study [14], because the thicknesses of the two specimens differed by more than two times.

Furthermore, ΔE* is quantified by the National Bureau of Standards (NBS), and ΔE* < 3.3 is considered a clinically acceptable value (NBS unit = ΔE* × 0.92) [40]. The Custom-block, PMMA-disk, and Conventional PMMA used in this study did not meet this requirement. In some previous studies [22,41], ΔE* < 3.3 was achieved only with discoloring media such as coffee and green tea, but ΔE* > 3.3 was achieved for curry solutions and wine; therefore, the results of this study were considered valid. In addition, no significant difference was observed between the results of Custom-block and Conventional PMMA, which suggests that the staining of Custom-block did not lead to clinical problems. PMMA-disk showed the lowest discoloration because of the material used; the generation of internal bubbles was suppressed owing to sufficient heating and pressurization [14,15,16], leading to difficulties in embedding the pigment in this area.

In the present in vitro study, a denture base resin, which is often used clinically, was selected, and the material was maintained at a constant temperature during testing. Furthermore, many previous studies have evaluated the basic physical properties of such materials, and tests were conducted in the present study for measuring three properties, namely three-point bending, water sorption, and solubility tests, as well as staining tests, which were used to interpret the results. A post hoc power analysis was performed using analysis software (G* Power 3.1.9.4 software, Kiel University, Kiel, Germany). As the significance level was α = 0.05 in this study and the detection power 1-β was 0.8 or higher, the sample size used in this study was considered appropriate.

A limitation of this study was that the materials of each block were different. As this study involved in vitro experiments, the various oral conditions could only be simulated to a limited extent. Furthermore, the ISO 20795-1 standard recommends that the flexural test of the denture base material be performed in a water bath at 37 °C. However, in the present study, the specimens were stored in purified water at 37 °C for 50 h, following which the flexural test was performed in air. Some studies reported that the water immersion of denture base materials decreased their flexural strength and flexural modulus [42]. Therefore, when the flexural test is performed under water immersion according to ISO20795-1, the tendency of the flexural strength does not change, but the strength may decrease. Moreover, the flexural test was performed until the specimen broke under a constant static load; however, the results may differ when a more clinical repeated load test is performed. Although the specimens were smoothly polished, the clinical denture base surface is uneven, and thus, the staining tendencies may differ. Therefore, in the future, thermal cycling and abrasion tests will be required to investigate the long-term durability of these materials.

## 5. Conclusions

Based on the results obtained in this study, the following conclusions can be drawn.

1. Significant differences in flexural strength were observed among the materials (*p* < 0.001). The flexural modulus and water solubility were significantly higher in the Custom-block. Furthermore, the water sorption of the Custom-block was significantly higher than that of the PMMA-disk, but it did not significantly differ from that of the Conventional PMMA. The discoloration of all the materials tended to increase initially, and till 7 days after the immersion.
2. With the exception of the staining test, the three materials met the ISO standard requirements for all tests, but the mechanical properties of the three materials differed depending on the manufacturing method used, which considerably affected the flexural strength, flexural modulus, water sorption, water solubility, and discoloration.

## Figures and Tables

**Table 1:** Denture base materials tested.

Material Name	Code	Polymer Type	Composition	Manufacturer	Curing
Fit resin	Custom-block	Pour-type PMMA Self-polymerizing resin	Powder: Copolymer of methyl methacrylate and 2-ethylhexyl acrylate, reaction initiator, coloring material, others Liquid: methyl methacrylate, ethylene glycol dimethacrylate, reaction initiator, others	Shofu, Japan	Self-polymerization at 50 °C for 30 min
Lucitone 199 denture base disc	PMMA-disk	CAD/CAM PMMA-based polymer	N/A	Dentsply Sirona, USA	N/A
Acron	Conventional PMMA	Conventional PMMA Heat-polymerized resin	Powder: Methacrylic acid ester polymer, others Liquid: Methyl methacrylate, others	GC, Japan	Heat curing at 78 °C for 8 h

**Table 2:** Flexural strength (MPa), flexural modulus (GPa), water sorption (μg/mm^3^), water solubility (μg/mm^3^), and discoloration (ΔE*) of the denture base materials calculated using Equations (1)–(5).

	Custom-Block	PMMA-Disk	Conventional PMMA
	Mean (SD)	Mean (SD)	Mean (SD)
Flexural strength (*FS*, MPa)	95.1 (4.3) ^a^	105.1 (2.2) ^b^	87.9 (5.0) ^c^
Flexural modulus (*FM*, GPa)	3.0 (0.1) ^d^	2.8 (0.1) ^e^	2.8 (0.0) ^e^
Water sorption (*W_sp_*, µg/mm^3^)	28.5 (2.6) ^fh^	23.2 (0.5) ^g^	25.6 (2.1) ^gh^
Water solubility (*W_sl_*, µg/mm^3^)	3.1 (16) ^i^	0.2 (0.1) ^j^	0.3 (0.2) ^j^
Discoloration (∆E*)	6.0 (0.6) ^k^	4.1 (0.8) ^l^	5.8 (0.4) ^k^

Values with the same superscript letter of ^a–l^ are not significantly different (*p* < 0.05).

## Data Availability

The data presented in this study are available on request from the corresponding author.
